# Dynamic changes in human brain connectivity following ultrasound neuromodulation

**DOI:** 10.1038/s41598-024-81102-w

**Published:** 2024-12-03

**Authors:** Cyril Atkinson-Clement, Mohammad Alkhawashki, Marilyn Gatica, James Ross, Marcus Kaiser

**Affiliations:** 1 Precision Imaging, School of Medicine, University of Nottingham, Nottingham, UK; 2 NIHR Biomedical Research Centre, University of Nottingham, Nottingham, UK; 3 NPLab, Network Science Institute, Northeastern University London, London, UK; 4 Rui Jin Hospital, Shanghai Jiao Tong University, Shanghai, China

**Keywords:** Focused ultrasound stimulation, Seed-based connectivity, Whole brain, Non-invasive neuromodulation, Motor inhibition, Stop signal task, Cognitive neuroscience, Motor control, Neural circuits

## Abstract

Non-invasive neuromodulation represents a major opportunity for brain interventions, and transcranial focused ultrasound (FUS) is one of the most promising approaches. However, some challenges prevent the community from fully understanding its outcomes. We aimed to address one of them and unravel the temporal dynamics of FUS effects in humans. Twenty-two healthy volunteers participated in the study. Eleven received FUS in the right inferior frontal cortex while the other 11 were stimulated in the right thalamus. Using a temporal dynamic approach, we compared resting-state fMRI seed-based functional connectivity obtained before and after FUS. We also assessed behavioural changes as measured with a task of reactive motor inhibition. Our findings reveal that the effects of FUS are predominantly time-constrained and spatially distributed in brain regions functionally connected with the directly stimulated area. In addition, mediation analysis highlighted that FUS applied in the right inferior cortex was associated with behavioural alterations which was directly explained by the applied acoustic pressure and the brain functional connectivity change we observed. Our study underscored that the biological effects of FUS are indicative of behavioural changes observed more than an hour following stimulation and are directly related to the applied acoustic pressure.

## Introduction

Low-intensity transcranial focused ultrasound stimulation (FUS) is a recent and high-potential neuromodulation approach^1^. While transcranial magnetic stimulation (TMS) and transcranial direct current stimulation (tDCS) can only reach cortical areas with low spatial specificity, FUS has several advantages. This method allows altering brain function safely^2,3^, with high spatial accuracy^4^, to reach deep brain areas, and potentially offer the choice of increasing or decreasing brain activity^5,6^. During the past decade, numerous animal studies have emphasised the potential of FUS to safely alter brain function^7–10^, paving the way for human experimentation.

Therefore, the number of human-based studies is rapidly growing. Many of them enlighten the processes which underpin FUS, revealing that FUS could increase cortical excitability^11–15^ through GABA inhibition^16^, that brain activity in the target region could be either increased^12,17–21^ or decreased^22^, that FUS could altered functional connectivity^13,16,17,23,24^ or even increase NMDA-dependent synaptic plasticity^25^. Meanwhile, others research already moved into more complex research on behavioural changes, including sensory discrimination^18,26–28^, motor inhibition^11,29–31^, reaction time^32^, approach and withdrawal behaviours^33^, mood^24^, pain^34^ and fear^35^. Last, the more audacious focused on pathological conditions such as epilepsy^36–38^, Alzheimer’s disease^39^ and other dementia^40^, consciousness disorders^41^, tremor^42^, Parkinson’s disease^43^, depression^44^ and chronic pain^45^).

However, FUS remains a recent and developing technique for which several challenges have to be addressed to improve our understanding of the mechanisms involved. Among them, at least three primary challenges have to be addressed: (i) deciphering the role of the FUS parameters^5,6,15,32^; (ii) assessing the multifactorial nature of result variability^16,46^; (iii) unravelling the temporal dynamics of the FUS effects^47^.

The present comparative pre-vs-post study was specifically designed to tackle the third challenge: we carefully considered the delay between FUS application and its effects (i.e., with a dynamic and high temporal resolution approach) on the whole brain. We administrated and assessed its brain (i.e., functional connectivity changes) and behavioural (inhibition and speed processing alterations) impacts before and right after the stimulation, on two groups of healthy humans with two brain targets: the right inferior frontal cortex (IFC) and the right thalamus. These two targets were chosen based on two criteria. First, their varying depths. First, their varying depths. The IFC is a cortical area, while the thalamus is one of the deepest brain structures and can only be reached non-invasively with FUS. Second, they have different functions. The IFC is well known for being involved in several cognitive domains, mostly related to motor inhibition^48^ through its direct connection with the subthalamic nucleus^49^ in the cortico-subthalamo-pallidal hyperdirect pathway^50^. The thalamus is more involved in motor and sensory mechanisms, but not specifically in motor inhibitions. Therefore, we expect a greater impact of IFC-FUS than Thal-FUS on motor inhibition outcomes.

As for every active approach, considering time allows to quantify the onset of an effect, its intensity and duration^51^. To date, most offline (i.e., brain and/or behavioural data obtained after the stimulation) human studies were conducted with insufficient consideration for the delay between FUS and the measured outcomes. As a result, such studies may overlook short-duration effects or misinterpret purely transient effects. Combined, our results highlight the time-constrained effects of FUS, as well as a relation between the applied pressure on the target, change in brain functioning and altered behavioural performances.

## Methods

### Participants

Twenty-two healthy, right-handed volunteers participated in the study (see Table 1, Table A1). They had no history of neurological or psychiatric disorders (excluding history of depression if considered as remitted from at least 1 year) and were not taking any medication. To mitigate potential risks associated with FUS and MRI, the following exclusion criteria were also applied: close relatives with a history of seizure, predisposition for syncope, excessive hair that could interfere with transducer coupling, current or planned pregnancy, implanted metallic device, skin disease, claustrophobia or anxiety related to MRI, tattoo near the head. Participants were instructed not to consume recreational drugs within 48 h before their visits and to limit alcohol intake to no more than four units within the preceding 24 h. Upon completion of the study, participants received compensation totalling £40, equivalent to £8 per hour.

**Table 1:** Demographic, experimental and acoustic properties of the two groups.

	Group IFC	Group Thalamus	*p*-values
Demographic			
Sex [F / M]	6 / 5	6 / 5	1
Age [years]	25 ± 5.7	22.8 ± 5.7	0.38
Sessions			
Session 1 [time of the day]	12h11 ± 2h46	14h00 ± 2h50	0.14
Session 2 [time of the day]	12h04 ± 2h57	13h59 ± 3h00	0.15
Delays			
Delay FUS rs-fMRI [min.sec]	15.00 ± 2.16	15.04 ± 1.37	0.95
Delay FUS SST [min.sec]	69.47 ± 7.28	70.08 ± 9.23	0.92
Acoustic simulation			
Target depth [mm]	31.1 ± 5	73.8 ± 3.7	**< 0.001**
ISPTA [mW/cm^2^]	83.3 ± 37.3	154.9 ± 69.2	**0.008**
MI	0.224 ± 0.061	0.298 ± 0.067	**0.013**
Maximum temperature increase [°C]	0.214 ± 0.09	0.393 ± 0.07	**< 0.001**
Peak pressure [kPa]*	153.5 ± 34.9	134.8 ± 26.7	0.173
Pek pressure in target [kPa]*	124.9 ± 57.4	103.3 ± 24.6	0.272
Activated volume [mm^3^]*	279.6 ± 147.2	1574.9 ± 689.5	**< 0.001**
Activated volume in target [mm^3^]*	85.4 ± 78.5	172.2 ± 91.4	**0.027**
Target activated [%]*	9.2 ± 8.5	18.6 ± 9.9	**0.027**
Activated volume in target [%]*	29.8 ± 21.7	11.3 ± 4.4	**0.018**

Values are given as mean ± standard deviation; bold values correspond to significant differences; * refers to values obtained after native to MNI transformation.

F, Female; IFC, inferior frontal cortex; ISPTA, spatial-peak temporal average intensity; M, male; MI, mechanical index; SST, stop signal task.

The study followed the ethical standards of the Helsinki Declaration of 1978, as revised in 2008, and was approved by the Ethics Committee of the University of Nottingham Faculty of Psychology (refence: F1298R). All participants gave their informed written consent for use of their data for research purposes.

### Study design

**Fig. 1:**
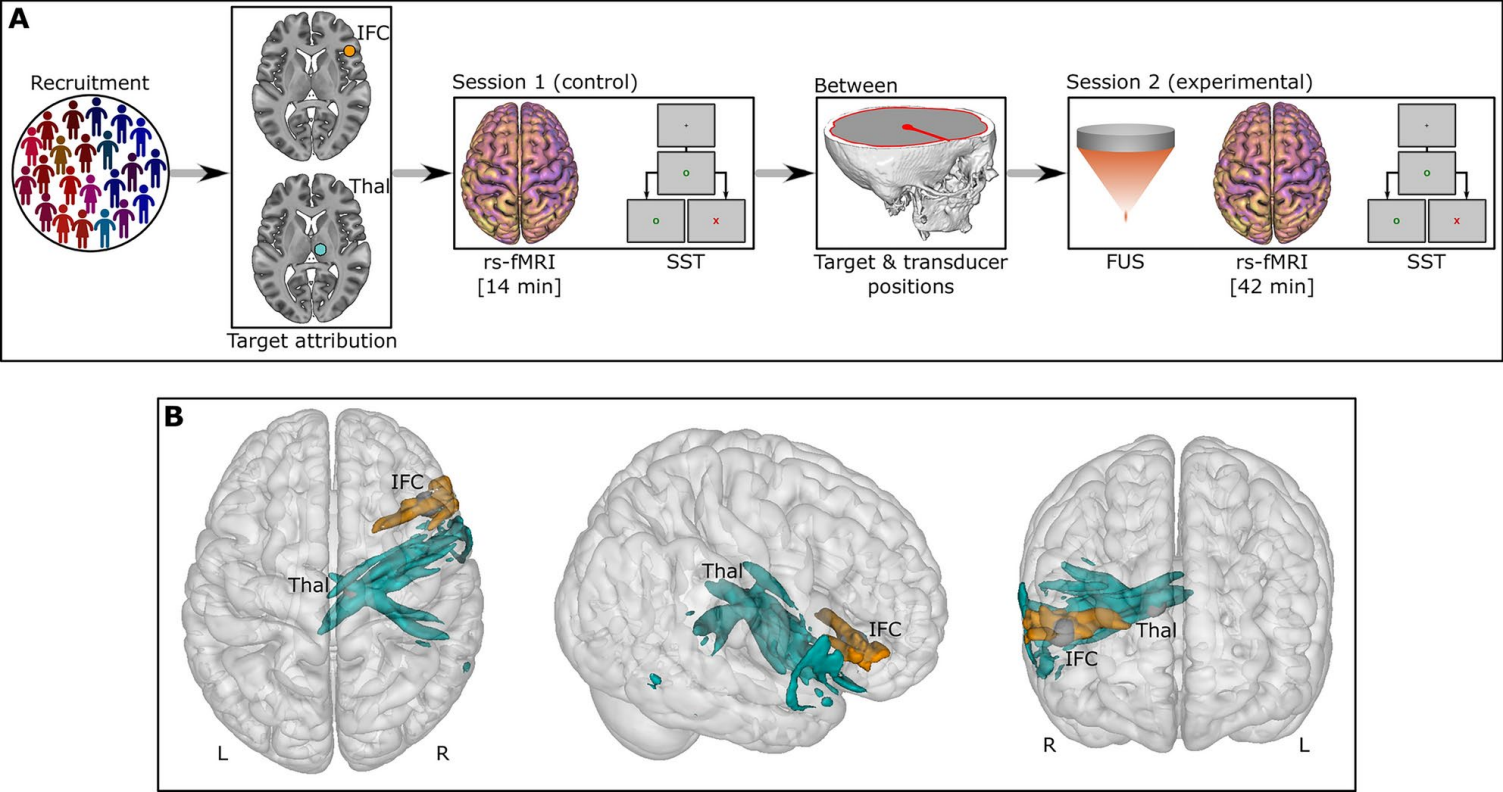
Study design. (**A**) 22 participants were recruited and then divided into two groups according to the brain target (IFC or Thal). The first session (“control”) consisted of an MRI protocol and a cognitive task. Next, the “between” section refers to the data preprocessing to identify the target and transducer positions. Finally, the second session (“experimental”) involved to the actual FUS followed by an MRI and a cognitive task. (**B**) Representation of the activated volume based on the acoustic simulations for all participants. Orange refers to the 11 participants with IFC-FUS whilst turquoise refers to the 11 participants with the Thalamus-FUS. IFC, inferior frontal cortex; L, Left; R, Right; rs-fMRI, resting-state functional MRI; SST, stop signal task; Thal, thalamus.

The study was organised into three phases, including two visits for the participants, as detailed below (Fig. 1, panel A).

Primarily, a first visit was scheduled during which the participants came to the University of Nottingham—Precision Imaging Beacon—United Kingdom. Here, we reviewed the inclusion and exclusion criteria before obtaining written consent. Then, they underwent a 45-min MRI session (which included a 14-min resting-state functional MRI [rs-fMRI] sequence), followed by a 15-min cognitive task performed outside the scanner.

After this visit, the participants were pseudo-randomly assigned to one of two groups (i.e., to ensure an equal distribution of sex; so-called stratified randomisation) corresponding to the FUS brain target (i.e., the right IFC or the right thalamus). We used the acquired MRI sequences to precisely identify the location of each brain target and determined the optimal placement of the FUS transducer^52^.

Then, a second visit was scheduled and the participants returned (median of 33 days between visits), at the same time of day as the initial visit (time difference of: 55.4 ± 40.1 min [IFC group] vs 69.5 ± 48.9 min [Thalamus group]; *p* = 0.469). All the criteria were verified a second time. FUS was then administered to the selected brain target. Immediately following stimulation, participants underwent another 45-min MRI session (delay FUS—rs-fMRI: 15 ± 2.16 min for the IFC group vs 15.4 ± 1.37 min for the thalamus group; *p* = 0.95; whose 42-min for the rs-fMRI sequence) followed by the same 15-min cognitive task achieved during the first visit and performed outside the scanner (delay FUS—cognitive task: 69.47 ± 7.28 min after FUS for the IFC group vs 70.8 ± 9.23 min after FUS for the thalamus group; *p* = 0.92).

Participants were asked to report any symptoms they experienced between the end of the stimulation up to 1 week afterwards using a dedicated follow-up questionnaire.

The groups were built as follows: all the data obtained during the first visit (n = 22) were considered as control and compared to the two experimental conditions depending on the FUS target, i.e., the right IFC (n = 11) and the right Thalamus (n = 11).

### MRI data, pre-processing and analysis

The full details of the MRI processing are available in supplementary materials. In short, the MRI scans were conducted using a General Electric 3 Tesla scanner. We compared the rs-fMRI obtained during the first session (14 min) to the rs-fMRI obtained right after the FUS (42 min). The other sequences were used for MRI preprocessing, acoustic simulations, transducer placement over the participants’ heads, and estimation of seed-based structural connectivity.

After preprocessing the data, we created dynamic maps to analyse seed-based connectivity using either the right inferior frontal cortex (IFC) or the right thalamus as the seed (see the section “Targets’ location” for more details). These maps were generated for each individual and session using a 100 TR window, moving by 1 TR. For the control condition (pre-FUS), we averaged the individual dynamic maps (n = 22; 499 volumes each) to obtain one map per participant, while for the experimental condition (post-FUS; 2 groups of 11; 1699 volumes each), we reduced the time dimensionality to obtain one map every minute following FUS. To do it, we aligned the individual maps to the delay following FUS and used a general additive model (GAM) to model the relationship between the delay and functional connectivity. Using a restricted maximum likelihood approach, the GAM determined the optimal degree of smoothing, and we selected the predicted functional connectivity values for every minute following FUS, considering only minutes fully covered by data (2 groups of 11; 42 volumes each). We then temporally aligned the dynamic maps of the participants to select the map corresponding to a specific delay following FUS in all participants. We considered only the time points for which all participants of the group (n = 11) had data available. The available observation windows were 20–47 min after FUS for the IFC group and 18–48 min after FUS for the Thalamus group. Finally, we compared the functional connectivity of the 22 controls to the 11 post-FUS using independent samples t-tests at every available time point. The significance threshold was set at *p* ≤ 0.05 for family-wise error rate (FWER) correction for the peak, and *p* ≤ 0.001 uncorrected for the neighbouring voxels. Clustering was performed on 4D maps (x, y, z, time).

### Cognitive task and analyses

The full details of the cognitive task are available in supplementary materials. Briefly, we used a computerised version of the stop signal task (SST)^53^. The task involved 4 blocks. During the first one (10-trial), participants had to press as fast as possible the spacebar after the presentation of a green circle on the screen (Go signal), allowing us to measure their response time. The second one (10-trial) was to train the participants with real instructions. Following the presentation of the Go signal, participants had to press the spacebar promptly. However, in 30% of cases, the Go signal was followed by a red cross (Stop signal), signalling participants to refrain from pressing the spacebar. Then, two blocs of 100 trials each were performed by the participants. The delay between the Go and Stop signals (stop-signal delay, SSD) began at 250 ms and was adjusted by 25 ms increments according to participants’ success (+ 25 ms) or failure (− 25 ms) in inhibiting their actions. We calculated the mean response time for successful Go trials and the stop signal reaction time (SSRT) using the time integration method as previously detailed^53^. The SSRT could be interpreted as the time maximal time for which the system could cancel a motor command. It corresponds to the mean SSD minus the quantile of the reaction time corresponding to the proportion of failed Stop trials (more details are available in supplementary materials).

To analyse the data, we used linear mixed models. Then, significant effects were correlated with the rs-fMRI values found at the peak of each significant cluster (x, y, z, time) and the acoustic simulation outputs. Lastly, causal mediation analyses were estimated using non-parametric bootstrapping (n = 1000). The threshold of significance was set at *p* ≤ 0.05.

### Targets’ location and transducer position

The location of the right IFC and thalamus were obtained on the Montreal Neurological Institute (MNI) coordinates using the AAL atlas^54^ as follows: right IFC (x = 46, y = 26, z = 8); right thalamus (x = 11, y = − 18, z = 7; corresponding to the centroid of the whole thalamus).

Between the two visits, and for each participant, the target location was moved from the MNI to the native space by using the inverse warp map between the T1-weighted sequence and the MNI template, obtained after co-registration. The right IFC was estimated to be 31.1 ± 5 mm deep (range: 24–42 mm) while the right thalamus was 73.8 ± 3.7 mm deep (range: 69–82 mm).

In the meantime, the Zero echo time (ZTE) sequence was used to extract the skull of each participant as a binary map. We then applied a custom-built R code to calculate both the distance between the brain target and the head, as well as the skull thickness for every possible trajectory (~ 250 k). The transducer coordinates which provided the lowest skull thickness were selected and incorporated into the neuronavigation software (Visor2™ V2.7, AntNeuro, Hengelo, Netherlands) as a point to pass through during the stimulation.

### Ultrasound stimulation and acoustic simulations

To apply the ultrasound stimulation, we used the NeuroFUS PRO TPO-203 with the four-element CTX-500-4CH transducer (Sonic Concepts, Brainbox Ltd., Cardiff, United Kingdom). The theta-burst FUS protocol was used^12,16^ as follows: central frequency = 500 kHz, pulse duration = 20 ms, pulse repetition interval = 200 ms (i.e., duty cycle = 10%), total duration = 80 s. The I_SPPA_ was set at 54.51W/cm^2^. These parameters are in line with the safety guidelines^55,56^. To ensure effective coupling between the ultrasound transducer and the participant’s head, ultrasound transmission gel was applied directly to the head, followed by application to the transducer face, with meticulous attention given to removing any air bubbles manually.

The details of the acoustic simulations’ procedure are available in supplementary materials, while the combined activated volumes are shown in Fig. 1B.

## Results

### Group differences

No difference was found between the two groups in terms of sex (6 females and 5 males in each group) and age (IFC: 25 ± 5.7; Thalamus: 22.8 ± 5.7; *p* = 0.38). During the experimental session, the delay between FUS and the start of rs-fMRI did not differ between the two groups (*p* = 0.95) as well as between the FUS and the SST (*p* = 0.92). The applied peak pressure (*p* = 0.173) and the peak pressure at the target (*p* = 0.272) did not differ between the two groups (see Table 1, Table A1, Fig. A1 and Fig. A2). Conversely, we found group differences regarding the size of the activated volume (*p* < 0.001), the percentage of the target area which was activated (which could be considered as a sensitivity measure; *p* = 0.027), the percentage of the activated volume which was in the target (which could be considered as a specificity measure; *p* = 0.018), the I_SPTA_ (*p* = 0.008), the mechanical index (*p* = 0.013) and the maximum temperature increase (*p* < 0.001; see Table 1, Table A1). Figure 1B highlights the shapes of the activated volumes for all participants.

### Side effects

Three participants reported side effects occurring within one hour after their participation and were not reported any longer after: The first one (IFC group) reported dizziness, muscle spasms in the right eye corner, and a lack of coordination; the second participant (IFC group) reported small muscle twitches in the legs; the third participant (thalamus group) reported being confused by the sound during the MRI.

### Dynamic of brain connectivity alterations following FUS

Dynamic changes were assessed by temporally smoothing the dynamic maps of every participant (using general additive models) and by extracting one averaged map per minute and per participant. Then, we compared all these maps voxels by voxels to the static maps obtained before FUS (n = 22) by using t-tests corrected for Family-wise Error Rate (FWER). See supplementary materials for details.

**Fig. 2:**
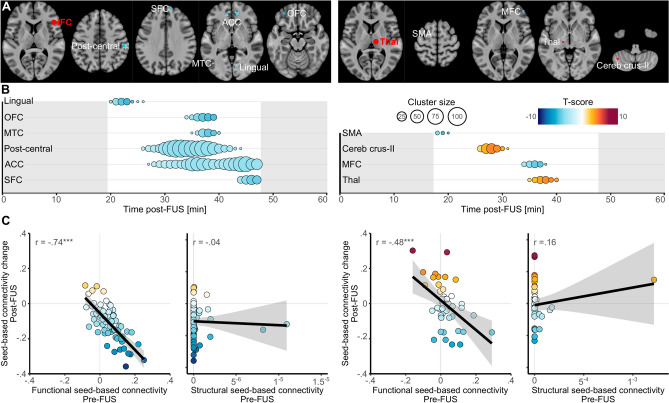
Functional connectivity changes following FUS. (**A**) Spatial locations of the significant clusters of seed-based connectivity changes following IFC-FUS (left) and Thalamus-FUS (right). The red sphere highlights the location of the target which was also used as seed. (**B**) Temporal locations of the significant clusters of seed-based connectivity changes following IFC-FUS (left) and Thalamus-FUS (right). The size of the dot reflects the size of the cluster (i.e., number of voxels) and the colours reflect the value of the peak at each moment (i.e., based on the T-score. A positive score indicates a significantly increased connectivity in comparison to the control condition, while a negative score indicates a decrease). (**C**) Correlations between the seed-based connectivity changes for each participant and cluster in the peak coordinates (in the 4 dimensions x, y, z, time) and the functional or structural seed-based connectivity for the IFC-FUS (left) and the Thalamus-FUS (right). ACC, anterior cingulate cortex; IFC, inferior frontal cortex; MFC, middle frontal cortex; MTC, middle temporal cortex; OFC, orbitofrontal cortex; SFC, superior frontal cortex; SMA, supplementary motor area; Thal, Thalamus.

Following IFC-FUS, we identified six different clusters in which the functional connectivity with the right IFC was decreased in comparison to the one acquired during the control session (n = 22; see Fig. 2, panels A and B, Tables A2, A3 and Fig. A6), involving: the right post-central cortex (from 26 to 44 min after FUS; total size: 236 voxels); the medial anterior cingulate cortex (ACC; 27–47 min; total size: 209 voxels); the medial superior frontal cortex (SFC; 44–47 min; total size: 29 voxels); the left middle temporal cortex (MTC, 35–40 min; total size: 24 voxels); the left inferior orbitofrontal cortex (OFC; 34–39 min; total size: 21 voxels); the right lingual cortex (20–26 min; total size: 17 voxels; details of the peak values before and after FUS are available in supplementary materials).

Following Thalamus-FUS (see Fig. 2, panels A and B, Tables A2, A3 and Fig. A6), we identified two clusters with a decreased functional connectivity with the right Thalamus (the right middle frontal cortex [MFC], 34–38 min after FUS, total size: 16 voxels; the left supplementary motor area [SMA], 18–20 min, total size: 4 voxels) and two clusters with increased functional connectivity (the left cerebellum Crus-II, 26–31 min, total size: 36 voxels; the left Thalamus, 35–40 min, total size: 15 voxels; details of the peak values before and after FUS are available in supplementary materials).

Interestingly, we found some clusters as being significantly changed at the beginning of our observation window (lingual cortex for IFC-FUS and SMA for Thalamus-FUS) while others remained significant at the end of our observation window (ACC and SFC for IFC-FUS).

When the comparisons were achieved on the static maps, no significant clusters reached our significance threshold. The detailed of the statistical values could be found in the Tables A2 and A3. Additionally, we found no significant difference between our two groups before FUS (independent two-sample t-tests) when FWER correction was applied. Based on uncorrected *p*-values threshold at *p* ≤ 0.001 with a cluster size of at least 10 voxels, only one significant difference was observed with a lower Thalamus-Cerebellum functional connectivity for the group Pre-Thal-FUS in comparison to the Pre-IFC-FUS group (x = 49, y = − 53, z = − 41, k = 10, t = 5.5, *p* = 0.00002). However, this cluster was not found as relevant following FUS. Due to the longer duration of the rs-fMRI sequence in the post-FUS condition in comparison to the pre-FUS condition, additional analysis on respiratory covariate was performed. No sign of sleepiness was observed (see supplementary materials).

### FUS effects occur in functionally connected parts of the brain

For both IFC and Thalamus FUS (see Fig. 2, panel C), we found that the degree of functional connectivity changes (by taking the coordinate of the peak for each cluster) was correlated with the strength of functional connectivity observed during the control condition (IFC: t_(64)_ = − 8.83, r = − 0.74, *p* < 0.001; Thalamus: t_(42)_ = − 3.59, r = − 0.48, *p* < 0.001). However, no significant relation was found with regards to structural connectivity (IFC: t_(64)_ = − 0.33, r = − 0.04, *p* = 0.74; Thalamus: t_(42)_ = 1.03, r = 0.16, *p* = 0.31).

### IFC-FUS changes behaviour

**Fig. 3:**
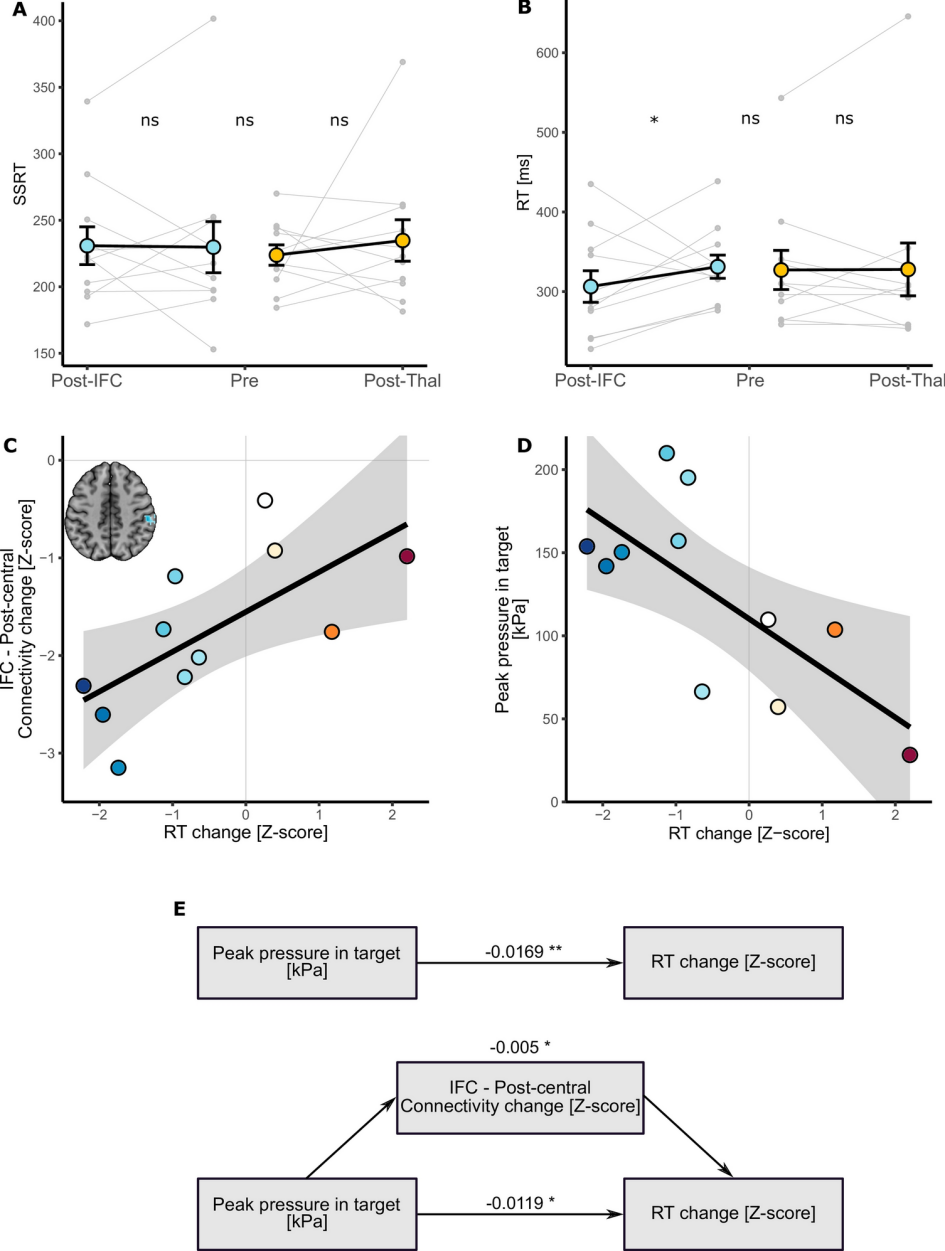
Behavioural changes following FUS. (**A**) No reactive inhibition (as measured by the SSRT) was found neither between groups nor after FUS. (**B**) Decreased response time was observed after IFC-FUS while no change was found after Thalamus-FUS. (**C**) Decrease in response time was significantly correlated with the decreased functional connectivity between the IFC and the post-central cortex. (**D**) Decrease in response time was also significantly correlated with the peak pressure estimated in the IFC. (**E**) The direct effect of the peak pressure applied to the IFC on the decrease in response time (top) was significantly mediated by the decreased functional connectivity between the IFC and the post-central cortex (bottom). The values correspond to the β of each effect. IFC, inferior frontal cortex; Post, refers to the experimental session, following FUS; Pre, refers to the control session, without FUS; RT, response time; SSRT, stop signal reaction time; Thal. Thalamus; ns, not significant; *p < 0.05; **p < 0.01.

Regarding behavioural changes following FUS (see Fig. 3, panels A and B) we found no significant alteration on the SSRT, for both IFC (t_(32)_ = − 0.015, *p* = 0.99; when removing the outlier: t_(29)_ = 0.73, *p* = 0.47) and Thalamus (t_(32)_ = 0.838, *p* = 0.4) groups. Conversely, we observed that IFC-FUS (n = 11) significantly decreased the RT performed during the “GO” trials in comparison to the control session (n = 22; t_(32)_ = − 2.011, *p* = 0.044), while Thalamus-FUS (n = 11) was not related to any change regarding RT (n = 22; t_(32)_ = 0.349, *p* = 0.73; when removing the outlier: t_(29)_ = − 1.026, *p* = 0.31).

Two significant correlations were found between the RT change (z-scored) and: (i) the decrease of functional connectivity between the IFC and the post-central cortex (r = 0.68, t_(9)_ = 2.82, *p* = 0.019; z-scored); (ii) the peak pressure observed in the target (r = 0.71, t_(9)_ = − 3.01, *p* = 0.015; see Fig. 3, panels C and D).

The results of the mediation analysis showed both significant direct and mediated effects (29.49% of the total; see Fig. 3, panel E) of the peak pressure on the behavioural change as follows: total effect: β = − 0.0169 [− 0.0298; − 0.01], *p* = 0.002; direct effect: β = − 0.0119 [− 0.0237;0], *p* = 0.012; indirect effect: β = − 0.0049 [− 0.0119;0], *p* = 0.05.

## Discussion

In this study involving two groups of 11 healthy humans each, we applied FUS to two brain targets, one cortical (i.e., IFC) and one deep (i.e., Thalamus). Our study is especially unique due to the temporal dynamic approach we used (i.e., one comparison between the control [n = 22] and the FUS [n = 11] conditions performed at every minute following the stimulation). Our findings emphasise that: (i) FUS effects are mainly time-constrained and connected to reduced functional connectivity with the parameters used; (ii) FUS causes connectivity alterations in the functional networks of the target, relating to the cerebello-thalamo-cortical network following Thalamus-FUS and to multiple networks changes after IFC-FUS; (iii) IFC-FUS is directly associated with behavioural alterations through the acoustic pressure applied to the IFC and the IFC-post-central cortex disconnection.

This study was innovative in that we addressed one of the ongoing challenges which prevent the community from fully understanding FUS outcomes: the significance of the delay between the stimulation and its effects. We addressed it with an innovative whole-brain and high time-resolution approach, on healthy humans and two different brain targets. With substantial sample sizes (two groups of 11) and considerable rs-fMRI scan duration (42 min), particularly notable in human FUS research. As one of the main outcomes, we identified a significant interrelation between the applied acoustic pressure, brain connectivity changes and behavioural alterations. This could lead to a more systematic use of FUS in tailored protocols which aim to change behaviour with a high degree of replicability.

However, some limitations deserve to be mentioned. First, we compared our experimental sessions to our control one, which is a post vs pre-FUS comparison, without a proper placebo (i.e., sham stimulation). If this is a weakness, it is worth noting that there is no validated placebo for FUS studies. Simply not turning on the transducer is not sufficient, as there are no associated sensory experiences (such as sounds and vibrations). Also, flipping over the transducer could cause damage, and targeting a different part of the brain could result in similar outcomes. Despite this, our findings remain robust because: (i) we used a stringent high significance threshold (i.e., Family-wise error rate correction) which reduces the possibility of false discovery; (ii) our outcomes are specific to each brain target group, indicating that they arise from FUS rather than participant awareness; (iii) we controlled for intra-individual differences by assessing the same participant at the same time of the day; and (iv) we controlled for inter-individual differences by building our IFC- and Thalamus-FUS groups to obtain similar sex ratios and ages. Second, we could argue that differences in the parts of the brain reached for each participant may have affected our results (see Fig. 1, panel B, Fig. A1 and Fig. A2). While FUS spatial accuracy surpasses other non-invasive neuromodulation methods, stimulation effects are not restricted to the target area, especially for deep targets. Depending on the used trajectory, the depth of the target and the skull thickness, the consequences of FUS could be different. It is not possible to determine this kind of effect, except through acoustic simulations, which are also mathematical estimations and not real measures. This is, to date, an inherent limitation to using FUS, and requires the development of more accurate devices and methodologies in the future. Third, the rs-fMRI were obtained ~ 15–20 min following FUS. We likely missed immediate and short-term effects. Future studies should consider performing online rs-fMRI with FUS to address this issue.

Our primary finding is about the onset and duration during which brain connectivity was altered by FUS. Altogether, our results highlight that brain connectivity was changed in a time-constrained manner which suggests a chain reaction manifesting by a spreading of the FUS effects. Indeed, whilst most of the clusters exhibited transient effects lasting only a few minutes, some persisted near our observation window borders, hinting at potentially longer-lasting effects (lingual cortex, ACC and SFC following IFC-FUS and SMA following Thalamus-FUS), and two were found to have a significantly longer duration (post-central cortex and ACC following IFC-FUS). This underlines the temporary yet significant effect of FUS, and it also reinforces the conclusion that a single 80-s stimulation session is enough to induce changes in brain function for a significant period of time^16^. Also interesting, most of the effects seem to be significant after a significant delay following FUS. This result is supported by an in vitro study which reported an increased in the mean frequency of evoked action potentials which was more significant 4–6 h after FUS than 0–2 h after^57^. To some extent, future studies could address the question of how many sessions are needed to induce permanent or long-term changes.

A second significant outcome is the strong relation we observed between FUS effects and the functional connectivity collected during the control condition, but not the structural connectivity (see Fig. 2, panel C). This result is promising in that it means that FUS consequences could reasonably be anticipated to occur within a known brain network. By learning from older neuromodulation approaches such as deep brain stimulation, we know that treatment should be individualised based on various data, including brain connectivity profiles^58,59^. Future studies will have to identify biomarkers related to FUS outcomes and then develop reliable models to maximise clinical improvement while mitigating adverse effects. We noticed that in this relationship, pre-FUS positive functional connectivity decreased, while negative pre-FUS positive functional connectivity increased. Overall, this led to the functional connectivity becoming closer to 0 following FUS. One possible interpretation of these result is that FUS did not have either an excitatory or inhibitory effect, but instead added noise to the system, resulting in an overall functional disconnection.

As evident in our results, Thalamus-FUS modulates the functioning of the cerebello-thalamo-cortical network in a time-distributed way: decreased connectivity with the left SMA (18–20 min post-FUS); increased connectivity with the contralateral cerebellum (Crus-II part; 26–31 min post-FUS); increased connectivity with the contralateral thalamus (35–40 min post-FUS). This result is of particular importance since the cerebello‑thalamo‑cortical network is known to be implicated in various brain conditions, involving numerous movement disorders^60^, schizophrenia^61^ and several neurodevelopmental disorders related to motor abnormalities^62^. Since the role of this network is precisely documented, many studies have already used non-invasive neuromodulation to alter its functioning^63,64^. Recently, a FUS study was conducted on 10 patients suffering from essential tremor^42^. Following Thalamus-FUS, the authors reported a significant clinical improvement in all participants. While this study was mostly a pilot and did not report any neuroimaging results, our findings support it and reinforce the relevance of targeting the thalamus to improve disorders involving the cerebello-thalamo-cortical network.

Regarding IFC-FUS, we exclusively found decreased connectivity, suggesting a functional disconnection of the IFC. This result contributes to the ongoing debate about which parameters induce excitatory or inhibitory effects. On the one hand, our findings are consistent with some articles which suggest that the parameters we used could be related to an inhibitory effect^5,6^. But on the other hand, our findings negate a recent study which used the same parameters as us, and found an excitatory effect as elicited by a decreased GABA concentration in one of the targets (i.e., posterior cingulate cortex), but not in the second (i.e., dorsal anterior cingulate cortex) and an overall increased functional connectivity^16^. However, as stated by the latter^16^, FUS effects could be state or location-dependent.

While half of our connectivity changes are in the anterior part of the brain (i.e., OFC, SFC and ACC), others are more spatially distributed. Based on the known functional connectivity of the IFC, all the clusters we found are in parts of the brain which are known to be related to the IFC^65^. In detail, our results are distributed among functional networks involved in reasoning (i.e., OFC, SFC and ACC clusters), motor execution (i.e., post-central cluster), social cognition (i.e., MTC cluster) and spatial attention (i.e., lingual cluster)^65^. This high variability could be explained by a lack of spatial accuracy regarding the activated volume by FUS (see Fig. 1, panel B).

Regarding the behavioural effects of IFC-FUS, two findings could be reported. On the one hand, our results do not align with our initial behavioural hypothesis, which was to induce an inhibitory process alteration following IFC-FUS (i.e., as measured by the SSRT). This discrepancy may be attributed to our inability to consistently target the specific subregion of the IFC involved in inhibitory processes, namely the posterior-ventral part^48^. Previous studies exploring the impact of FUS on reactive inhibition have yielded mixed outcomes, as inhibitory process changes were not always observed: improved only when IFC-FUS was applied during STOP trials^29^; decreased SSRT following M1-FUS.

On the other hand, we induced a significant reduction in the response time for the GO trials which was explained by the acoustic pressure applied to the IFC and by the functional disconnection between the right IFC and the post-central cortex (see Fig. 3, panels C, D and E). Importantly, the absence of inhibitory changes associated with this speed improvement suggests that participants did not adjust their behaviour (i.e., going faster despite increased risk of failing STOP trials). Furthermore, this effect could not be attributed to a learning effect, as it was not observed following Thalamus-FUS.

One plausible explanation could be that the disconnection of executive and associative networks (involved in inhibition)^50^, through IFC-FUS, did not influence reactive inhibition (as measured by the SSRT) but decreased tonic proactive inhibition played by the IFC to the post-central cortex^66^, and therefore released the ability to do a motor action faster. This interpretation is consistent with previous observations of improved response speed following IFC-tDCS^67^ or M1-FUS^32^ and aligns with the established role of the IFC in both reactive and proactive inhibition^66^.

## Conclusions

This study contributes to unravelling FUS mechanisms and brings findings that collectively enhance our comprehension of FUS effects on humans. By addressing the time dynamics of seed-based functional connectivity alterations following FUS on two different brain targets, we underscore that the majority of FUS effects are time-constrained, occur within functionally connected brain regions, span across one or multiple functional networks, and could change behavioural performance for at least an hour after post-stimulation.

Research on human-FUS neuromodulation is beginning, further studies are imperative to safely and responsibly use it in clinical populations. Among them: what is the combination of parameters to elicit either excitatory or inhibitory effects? What are the immediate and short-term effects of FUS and what is their spatiotemporal spread? What number of sessions is needed to induce long-term changes? How predictable are FUS outcomes at the individual level?

## Supplementary Information

Supplementary Information 1.

Supplementary Information 2.

## Data Availability

All data and original code used to analyse the data in this paper are available from the corresponding contact upon request.
