# Supplementary material for: Dynamic changes in human brain connectivity following ultrasound neuromodulation
Source: Sci Rep. 2024 Dec 3;14:30025. doi: 10.1038/s41598-024-81102-w (PMC11614892; doi:10.1038/s41598-024-81102-w)
Supplement: Supplementary file 1 — Supplementary Information 1. [file 41598_2024_81102_MOESM1_ESM.docx]

**Dynamic Changes in Human Brain Connectivity Following Ultrasound Neuromodulation**

Cyril Atkinson-Clement^1,2*^, Mohammad Alkhawashki^1^, Marilyn Gatica^1^, James Ross^1^, Marcus Kaiser^1,2,3^

^1^ Precision Imaging, School of Medicine, University of Nottingham, United Kingdom.

^2^ NIHR Biomedical Research Centre, University of Nottingham, United Kingdom.

^3^ Rui Jin Hospital, Shanghai Jiao Tong University, Shanghai, China.

**Supplementary materials**

**MRI data**

MRI scans were conducted using a General Electric 3 Tesla scanner equipped with a 48-channel head coil. During the initial visit, the following protocol was employed:

1. T1-weighted magnetisation-prepared rapid gradient echo (MPRAGE; repetition time [TR]=2.282sec; echo time [TE]=2.96ms; inversion time [TI]=800ms; flip angle [FA]=8°; field of view [FOV]=256 x 256 mm; 180 slices of 1mm^3^ isotropic voxels);

2. Zero echo time (ZTE; TR=0.531sec; TE=0.016ms; FOV=256 x 256 mm; 176 slices of 1.016 x 1.016 x 1 mm^3^ voxels);

3. Shimming (2 echoes; TE=4.54ms; FOV=240 x 240 mm; 32 slices of 3.75 x 3.75 x 5.8 mm^3^; direction=right to left);

4. Diffusion-weighted Imaging (DWI; TR=4.6sec; TE=90ms; 63 slices of 2.019 x 2.09 x 2 mm^3^; direction=right to left; b-values=0, 300, 1000, 2000 s/mm2 with respectively 0, 10, 50, 50 directions);

5. Resting-state functional MRI (rs-fMRI; 14-minute; TR=1.4sec; TE=35ms; FA=68°; FOV=212 x 212 mm; 88 interleaved slices of 2mm^3^ isotropic voxels; no slice gap; multiband acceleration factor of 3). Cardiac and respiratory data were registered during this sequence. This sequence was obtained with the eyes opened.

During the second visit, participants underwent the following MRI protocol:

6. Resting-state functional MRI (rs-fMRI; 42-minute; TR=1.4sec; TE=35ms; FA=68°; FOV=212 x 212 mm; 88 interleaved slices of 2mm^3^ isotropic voxels; no slice gap; multiband acceleration factor of 3). Cardiac and respiratory data were registered during this sequence.

7. T1-weighted magnetisation-prepared rapid gradient echo (MPRAGE; TR=2.282sec; TE=2.96ms; TI=800ms; FA=8°; FOV=256 x 256 mm; 180 slices of 1mm^3^ isotropic voxels). This sequence was obtained with the eyes opened.

**MRI pre-processing and analysis**

All preprocessing were achieved using AFNI[1], ANTs[2], Freesurfer (<http://surfer.nmr.mgh.harvard.edu/>), FSL[3], MRtrix[4] and R[5].

*ZTE*

The purpose of the ZTE sequence was to extract the skull shape of each participant, and then calculate the skull thickness to find the best FUS trajectory (see below). To do this, we first enhanced the contrast of the T1 image and then aligned the ZTE with the enhanced T1 image. The aligned T1 was used to extract the head and the brain (both as binary maps), enabling us to derive the ZTE values for both brain tissue and background. The skull was estimated to fall above ZTE+1SD of the brain tissue, and below ZTE-1SD of the background. Manual adjustments were made to these values as necessary.

*PCT*

For the acoustic simulations, the T1 images was converted to Pseudo-CT (PCT)[6] (<https://github.com/sitiny/mr-to-pct>).

*DTI*

DTI data were pre-processed using the standard approach provided by MRtrix, including denoising, removal of Gibbs artefacts, FSL preprocessing, B1 field inhomogeneity correction, and unsupervised estimation of brain tissues' multi-shell multi-tissue fibre orientation distributions. Subsequently, one million tracts were estimated using a seed-based approach, where all tracts crossing the FUS target were extracted and moved as a proportion to the MNI space. This approach allowed us to build seed-based structural connectivity of every voxel connected to the FUS target.

*Rs-fMRI*

Rs-fMRI were pre-processed by first removing signals from the ventricles and white matter (extracted with Freesurfer; regressed by removing the first principal component) as well as cardiac and respiratory artefacts (collected during the sequence; with the AFNI 3dretroicor function). Then, we despike the data and applied the afni_proc.py script, which involved the following steps: blocks (discarding the first two volumes), tshift, align (with the minimum outlier volume), tlrc, volreg, mask, scale (each volume mean was set to 100 with a voxel maximum value of 200), regress (including motion, by removing the mean and derivatives in a regression model). No volume was removed. The MRI were co-registered to the MNI152 template (https://github.com/Jfortin1/MNITemplate).Then, a 4mm smoothing was applied.

After pre-processing, we generated dynamic maps for seed-based connectivity. We used a window of 100 TR, moving by 1 TR. To reduce time dimensionality, we used a general additive model (GAM) which automatically determined the optimal smoothing parameters with a restricted maximum likelihood approach. We then extracted predicted values for each minute, considering only minutes fully covered by data.

For the control condition (pre-FUS), we calculated the average of the dynamic maps. We then aligned the dynamic maps temporally among the 11 participants in each group and compared them to the data from the 22 controls using independent samples t-tests on each voxel and at every minute. The available observation windows were of 20-47 minutes after FUS for the IFC group and 18-48 minutes after FUS for the Thalamus group. The significance threshold was set at p ≤ 0.05 for Family-wise Error Rate (FWER) correction for the peak, and p ≤ 0.001 uncorrected for the neighbouring voxels.

Given that clustering was performed on 4D maps (x, y, z, time), we used a density-based spatial clustering (DBSCAN[7]) with a maximum Euclidean distance of 1.5 mm between two significant voxels (which corresponds to the share of at least one face). Similar analyses were conducted on static maps to evaluate the importance of using a dynamic approach.

**Cognitive task and analyses**

We used a computerised version of the Stop Signal Task (SST)[8,9] implemented in Matlab[10] through the Psychophysics toolbox extensions - version 3[11,12]. The task was presented on a Windows 11 laptop with a screen size of 17.25 inches and a refresh rate of 60.13Hz. The total duration of the task was approximately 15 minutes.

The task began with a 10-trial block during which the response time was measured. Participants were shown a white cross in the centre of the screen for a randomly varying duration between 1 and 2 seconds, followed by a green circle prompting them to press the spacebar as quickly as possible (Go signal). If the response time was longer than 2 seconds, the trial was disregarded. If the participant pressed the spacebar before the presentation of the Go signal, the trial was disregarded and feedback indicating that the participant needed to wait until the Go signal was presented was given. This block aimed to determine the mean and standard deviation (SD) of response times for each participant.

Subsequently, a 10-trial training block was performed. A white cross was displayed with a variable delay of 1 to 2 seconds, followed by the Go signal instructing participants to press the spacebar promptly. However, in 30% of cases, the Go signal was followed by a red cross (Stop signal), signalling participants to refrain from pressing the spacebar. The delay between the Go and Stop signals (stop-signal delay, SSD) began at 250ms and was adjusted by 25ms increments according to participants' success or failure in inhibiting their actions. If the participants successfully stopped their action, the SSD increased, making subsequent inhibition more challenging. Conversely, failure to inhibit action resulted in a decreased SSD, making the task easier.

After the training block, the task was divided into two blocks of 100 trials each, following the same parameters as previously specified. Each block started with a 250ms SSD. A point system was introduced to encourage task performance. If the participant pressed the spacebar prior to the presentation of the Go signal, 1 point was lost. During Go trials, if the participant hit the spacebar within the mean±1SD response time (acquired in the first block), 1 point was earned. If this delay was of mean±2SD response time, 0.5 points were earned. If this delay was longer, 0.5 points were lost. If the participant did not press the spacebar 2 seconds after the Go signal, 1 point was lost. During the Stop trials, if the participant successfully cancelled their actions, 1 point was earned, otherwise, 0.5 points were lost.

For the two blocks of 100 trials, we calculated the mean response time for successful Go trials and the Stop Signal Reaction Time (SSRT) using the time integration method as previously detailed[8,9] (<https://cran.r-project.org/web/packages/splithalfr/vignettes/sst_ssrti.html>). In detail, SSRT calculation requires the mean of the SSD, the proportion of failed Stop trials, and the response time distribution. Then, the SSRT corresponds to the mean SSD minus the quantile of the reaction time corresponding to the proportion of failed Stop trials (the full code is available on the weblink mentioned above).

Changes in response time and SSRT were analysed using linear mixed models[13]. Then, significant effects were correlated with the rs-fMRI values found at the peak of each significant cluster (x, y, z, time) and the acoustic simulation outputs. Lastly, causal mediation analyses were estimated using non-parametric bootstrapping (n=1000)[14]. The threshold of significance was set at p≤0.05.

**Acoustic simulations**

We used k-plan V1.0 (Brainbox Ltd., Cardiff, United Kingdom) for the acoustic simulations. They were carried out after data acquisition by using both the acquired T1-weighted and a pseudo-computed tomography (PCT) scans that were estimated for each participant from the T1-weighted scans[6]. The pressure estimation maps were then moved from the native to the template space by using the conversion map obtained during the MRI pre-processing. Every data point based on pressure information was derived from the values computed in the template space (see Table.1 and Table.A1 for details). The estimation of the activated volume (see Table.1, Table.A1 and Fig.1) was based on the full width at half maximum (FWHM) of the ultrasound beam (i.e., every voxel with a pressure higher than 50% of the maximum applied was considered as an activated volume). We also reported the percentage of the target which was activated, as well as the percentage of the activated volume which was in the target. The first could be considered as a sensitivity measure (ability to reach the target) while the second could be considered as a specificity measure (ability to only reach the target). This is required because the deeper the target, the larger the size of the activated volume, which is due to the ultrasound transducer angle and the effect of phased array steering of focus point depth. There is an inverse relationship between depth of focus and spatial resolution; deeper focus leads to lower resolution. This explains why the percentage of the target which was activated was higher in the deeper Thalamus group in comparison to the IFC group (p=0.027). It also explains why the size of the activated volume which was in the target was lower for the Thalamus group in comparison to the IFC group (p=0.018; see Table.1 and Table.A1). Acoustic pressure estimations for all participants are available in the Fig.A1 and Fig.A2.

**References**

[1] Cox RW. AFNI: Software for Analysis and Visualization of Functional Magnetic Resonance Neuroimages. Computers and Biomedical Research 1996;29:162–73. https://doi.org/10.1006/cbmr.1996.0014.

[2] Avants BB, Tustison NJ, Song G, Cook PA, Klein A, Gee JC. A reproducible evaluation of ANTs similarity metric performance in brain image registration. NeuroImage 2011;54:2033–44. https://doi.org/10.1016/j.neuroimage.2010.09.025.

[3] Jenkinson M, Beckmann CF, Behrens TEJ, Woolrich MW, Smith SM. FSL. NeuroImage 2012;62:782–90. https://doi.org/10.1016/j.neuroimage.2011.09.015.

[4] Tournier J-D, Mori S, Leemans A. Diffusion tensor imaging and beyond. Magn Reson Med 2011;65:1532–56. https://doi.org/10.1002/mrm.22924.

[5] R Core Team. R: A language and environment for statistical computing. Vienna, Austria: R Foundation for Statistical Computing; 2021.

[6] Yaakub SN, White TA, Kerfoot E, Verhagen L, Hammers A, Fouragnan EF. Pseudo-CTs from T1-weighted MRI for planning of low-intensity transcranial focused ultrasound neuromodulation: An open-source tool. Brain Stimulation 2023;16:75–8. https://doi.org/10.1016/j.brs.2023.01.838.

[7] Hahsler M, Piekenbrock M, Doran D. dbscan: Fast Density-Based Clustering with R. J Stat Soft 2019;91. https://doi.org/10.18637/jss.v091.i01.

[8] Logan GD, Cowan WB, Davis KA. On the ability to inhibit simple and choice reaction time responses: a model and a method. J Exp Psychol Hum Percept Perform 1984;10:276–91.

[9] Verbruggen F, Aron AR, Band GP, Beste C, Bissett PG, Brockett AT, et al. A consensus guide to capturing the ability to inhibit actions and impulsive behaviors in the stop-signal task. eLife 2019;8:e46323. https://doi.org/10.7554/eLife.46323.

[10] Matlab. 9.7.0.1190202 (R2019b). Natick, Massachusetts: The MathWorks Inc.; 2018.

[11] Brainard DH. The Psychophysics Toolbox. Spat Vis 1997;10:433–6.

[12] Pelli DG. The VideoToolbox software for visual psychophysics: transforming numbers into movies. Spatial Vis 1997;10:437–42. https://doi.org/10.1163/156856897X00366.

[13] Bates D, Mächler M, Bolker B, Walker S. Fitting Linear Mixed-Effects Models Using lme4. J Stat Soft 2015;67. https://doi.org/10.18637/jss.v067.i01.

[14] Tingley D, Yamamoto T, Hirose K, Keele L, Imai K. mediation: R Package for Causal Mediation Analysis. J Stat Soft 2014;59. https://doi.org/10.18637/jss.v059.i05.

**Table.A1. Demographic, experimental and acoustic properties of all participants.**

|  | **Demographic** | | **Visits** | | **Delay** | | **Acoustic simulation** | | | | | | | | | |
| --- | --- | --- | --- | --- | --- | --- | --- | --- | --- | --- | --- | --- | --- | --- | --- | --- |
| ID | Sex | Age | Visit 1  [time of the day] | Visit 2  [time of the day] | Delay  FUS-fMRI [min.sec] | Delay  FUS-SST [min.sec] | Target depth [mm] | ISPTA [mW/cm^2^] | MI | Maximum temperature increase  [°C] | Peak pressure [kPa]* | Peak pressure in target [kPa]* | Activated volume (mm^3^)* | Activated volume in target (mm^3^)* | Target activated (%)* | Activated volume in target  (%)* |
| **Group IFC** | | | | | | | | | | | | | | | | |
| P002 | M | 29 | 8h30 | 8h30 | 12.20 | 69.20 | 27 | 126 | 0.275 | 0.176 | 191.7 | 157.1 | 127 | 30 | 3.2 | 23.6 |
| P004 | F | 39 | 13h30 | 14h20 | 19.05 | 70.00 | 31 | 101 | 0.246 | 0.211 | 171.2 | 57.2 | 118 | 0 | 0 | 0 |
| P008 | M | 20 | 11h30 | 13h30 | 16.50 | 90.15 | 42 | 128 | 0.277 | 0.348 | 195.6 | 195.2 | 183 | 79 | 8.5 | 43.2 |
| P009 | F | 22 | 13h30 | 14h20 | 14.50 | 67.15 | 24 | 46.1 | 0.166 | 0.099 | 116.4 | 66.4 | 406 | 13 | 1.4 | 3.2 |
| P010 | M | 20 | 16h30 | 15h50 | 16.50 | 69.30 | 29 | 40.1 | 0.155 | 0.124 | 109.7 | 109.7 | 476 | 220 | 23.8 | 46.2 |
| P011 | F | 28 | 8h30 | 8h00 | 14.30 | 68.05 | 29 | 61.1 | 0.192 | 0.181 | 135.4 | 28.3 | 220 | 0 | 0 | 0 |
| P012 | M | 20 | 9h30 | 10h00 | 13.05 | 64.30 | 29 | 75.3 | 0.213 | 0.252 | 150.3 | 150.3 | 456 | 204 | 22 | 44.7 |
| P014 | F | 26 | 13h30 | 11h20 | 15.40 | 70.30 | 33 | 147 | 0.355 | 0.355 | 209.9 | 209.9 | 198 | 79 | 8.5 | 39.9 |
| P016 | F | 22 | 10h30 | 9h20 | 13.30 | 60.25 | 28 | 42.6 | 0.160 | 0.107 | 112.7 | 103.7 | 474 | 84 | 9.1 | 17.7 |
| P020 | F | 22 | 12h30 | 11h20 | 11.40 | 66.30 | 37 | 67.2 | 0.201 | 0.294 | 141.9 | 141.9 | 294 | 164 | 17.7 | 55.8 |
| P022 | M | 27 | 16h00 | 16h20 | 16.45 | 71.20 | 33 | 82.3 | 0.222 | 0.203 | 153.8 | 153.8 | 124 | 67 | 7.2 | 54 |
| **Mean** | **-** | **25** | **12h11** | **12h04** | **15.00** | **69.47** | **31.1** | **83.3** | **0.224** | **0.214** | **153.5** | **124.9** | **279.6** | **85.4** | **9.2** | **29.8** |
| **SD** | **-** | **5.7** | **2h46** | **2h57** | **2.16** | **7.28** | **5** | **37.3** | **0.061** | **0.09** | **34.9** | **57.4** | **147.2** | **78.5** | **8.5** | **21.7** |
| **Group Thalamus** | | | | | | | | | | | | | | | | |
| P005 | F | 19 | 14h00 | 16h00 | 13.25 | 66.20 | 69 | 136 | 0.285 | 0.456 | 118.9 | 110.5 | 1633 | 330 | 35.7 | 20.2 |
| P006 | F | 20 | 15h30 | 14h50 | 13.35 | 65.30 | 76 | 197 | 0.344 | 0.521 | 102.8 | 64.6 | 3221 | 220 | 23.8 | 6.8 |
| P007 | F | 21 | 8h30 | 8h00 | 15.20 | 66.30 | 77 | 284 | 0.413 | 0.467 | 162.2 | 112.5 | 1104 | 100 | 10.8 | 9.1 |
| P013 | F | 25 | 15h00 | 13h45 | 17.20 | 69.30 | 73 | 86.9 | 0.228 | 0.357 | 113.9 | 100.2 | 1679 | 203 | 21.9 | 13.7 |
| P015 | M | 19 | 16h00 | 14h20 | 15.00 | 97.50 | 75 | 230 | 0.371 | 0.365 | 106.9 | 70.6 | 1822 | 122 | 13.2 | 6.7 |
| P017 | M | 20 | 15h30 | 14h45 | 14.15 | 65.30 | 71 | 86.6 | 0.228 | 0.310 | 145.4 | 124.8 | 1072 | 158 | 17.1 | 14.7 |
| P018 | M | 18 | 8h30 | 8h30 | 15.00 | 67.50 | 71 | 124 | 0.273 | 0.439 | 184.5 | 123.3 | 731 | 69 | 7.5 | 9.4 |
| P019 | M | 27 | 14h30 | 16h45 | 12.45 | 65.40 | 82 | 220 | 0.364 | 0.363 | 110.3 | 71.2 | 1440 | 77 | 8.3 | 5.3 |
| P021 | F | 22 | 17h00 | 15h20 | 14.30 | 66.30 | 70 | 161 | 0.310 | 0.410 | 139.5 | 137.4 | 2206 | 326 | 35.2 | 14.8 |
| P023 | M | 22 | 15h00 | 17h00 | 17.40 | 71.30 | 74 | 78.3 | 0.217 | 0.307 | 136.7 | 101.5 | 1428 | 180 | 19.5 | 12.6 |
| P024 | F | 38 | 14h30 | 14h30 | 16.50 | 68.50 | 74 | 99.7 | 0.245 | 0.331 | 161.3 | 119.5 | 988 | 109 | 11.8 | 11 |
| **Mean** | **-** | **22.8** | **14h00** | **13h59** | **15.4** | **70.8** | **73.8** | **154.9** | **0.298** | **0.393** | **134.8** | **103.3** | **1574.9** | **172.2** | **18.6** | **11.3** |
| **SD** | **-** | **5.7** | **2h50** | **3h00** | **1.37** | **9.23** | **3.7** | **69.2** | **0.067** | **0.07** | **26.7** | **24.6** | **689.5** | **91.4** | **9.9** | **4.4** |
| **p-value** | **1** | **0.38** | **0.14** | **0.15** | **0.95** | **0.92** | **<0.01** | **0.008** | **0.013** | **<0.01** | **0.173** | **0.272** | **<0.01** | **0.027** | **0.027** | **0.018** |

Values are given as mean ± standard deviation; bold values correspond to significant differences; * refers to values obtained after native to MNI transformation.

F: Female; IFC: Inferior Frontal Cortex; ISPTA: Spatial-Peak Temporal Average Intensity; M: Male; MI: Mechanical Index; SST: Stop Signal Task.

**Table.A2. Significant seed-based connectivity alterations following FUS.**

| **Cluster size** | **Locations** | **Peak** | | | | | **Moment of significance [min post-FUS]** |
| --- | --- | --- | --- | --- | --- | --- | --- |
|  |  | **x** | **y** | **z** | **time** | **t-score** |  |
| **Group IFC** | | | | | | | |
| 236 | Post-central - R (57.3%)  Supramarginal - R (31.5%)  Inferior Parietal - R (9.5%)  Superior Parietal - R (1.7%) | 55 | -27 | 45 | 33 | -6.75 | 26-44 |
| 209 | Anterior Cingulum - L (36.1%)  Superior Frontal Medial - L (23.7%)  Superior Frontal Medial - R (20.1%)  Anterior Cingulum– R (10.8%)  Orbitofrontal Medial - R (7.7%)  Superior Frontal - L (1.5%) | -9 | 43 | 3 | 47 | -6.81 | 27-47 |
| 29 | Superior Frontal Medial - R (89.3%)  Superior Frontal - R (10.7%) | 11 | 57 | 33 | 47 | -6.75 | 44-47 |
| 24 | Middle Temporal - L (66.6%)  Middle Occipital - L (33.3) | -45 | -65 | 3 | 38 | -6.59 | 35-40 |
| 21 | Inferior Orbitofrontal - L (60%)  Middle Orbitofrontal– L (40%) | -31 | 47 | -17 | 38 | -8.02 | 34-39 |
| 17 | Lingual - R (100%) | 11 | -77 | -1 | 23 | -7.76 | 20-26 |
| **Group Thalamus** | | | | | | | |
| 36 | Cerebellum Crus-II - L (52.8%)  Cerebellum 7b - L (33.3%)  Cerebellum 8 - L (13.9%) | -41 | -57 | -49 | 26 | 6.76 | 26-31 |
| 16 | Middle Frontal - L (100%) | 47 | 49 | 5 | 35 | -6.54 | 34-38 |
| 15 | Thalamus - L (100%) | -15 | -19 | -3 | 38 | 6.65 | 35-40 |
| 4 | SMA - L (100%) | -9 | 1 | 63 | 19 | -6.95 | 18-20 |

Note: the labelling was based on the AAL atlas

**Table.A3. Details of the significant clusters for each minutes following FUS.**

| **ID** | **Time post-TUS** | **Total size** | **Locations** |
| --- | --- | --- | --- |
| **Group IFC** | | | |
| 1 | 26 | 3 | Supramarginal-R (3) |
|  | 27 | 11 | Supramarginal-R (10) - Inferior parietal-R (1) |
|  | 28 | 14 | Supramarginal-R (11) - Inferior parietal-R (2) - Postcentral-R (1) |
|  | 29 | 20 | Supramarginal-R (13) - Postcentral-R (5) - Inferior parietal-R (2) |
|  | 30 | 47 | Postcentral-R (32) - Supramarginal-R (14) - Inferior parietal-R (1) |
|  | 31 | 71 | Postcentral-R (55) - Supramarginal-R (15) - Inferior parietal-R (1) |
|  | **32** | **89** | Postcentral-R (61) - Supramarginal-R (26) - Inferior parietal-R (2) |
|  | **33** | **89** | Postcentral-R (49) - Supramarginal-R (38) - Inferior parietal-R (2) |
|  | 34 | 81 | Postcentral-R (39) - Supramarginal-R (39) - Inferior parietal-R (3) |
|  | 35 | 74 | Supramarginal-R (37) - Postcentral-R (29) - Inferior parietal-R (8) |
|  | 36 | 68 | Supramarginal-R (32) - Postcentral-R (22) - Inferior parietal-R (13) - Superior parietal-R (1) |
|  | 37 | 77 | Postcentral-R (38) - Supramarginal-R (21) - Inferior parietal-R (16) - Superior parietal-R (2) |
|  | 38 | 62 | Postcentral-R (39) - Supramarginal-R (10) - Inferior parietal-R (9) - Superior parietal-R (4) |
|  | 39 | 42 | Postcentral-R (28) - Inferior parietal-R (6) - Supramarginal-R (6) - Superior parietal-R (2) |
|  | 40 | 24 | Postcentral-R (20) - Inferior parietal-R (2) - Supramarginal-R (2) |
|  | 41 | 13 | Postcentral-R (13) |
|  | 42 | 9 | Postcentral-R (9) |
|  | 43 | 2 | Postcentral-R (2) |
|  | 44 | 1 | Postcentral-R (1) |
| 2 | 27 | 1 | Medial superior frontal-R (1) |
|  | 28 | 7 | Medial superior frontal-R (7) |
|  | 29 | 13 | Medial superior frontal-R (13) |
|  | 30 | 17 | Medial superior frontal-R (15) – Medial orbitofrontal-R (1) – Anterior cingulate-L (1) |
|  | 31 | 19 | Medial superior frontal-R (11) – Medial superior frontal-L (3) – Medial orbitofrontal-R (2) – Anterior cingulate-L (2) |
|  | 32 | 20 | Medial superior frontal-L (5) – Medial orbitofrontal-R (5) – Anterior cingulate-L (4) – Medial superior frontal-R (3) – Anterior cingulate-R (3) |
|  | 33 | 19 | Medial superior frontal-R (6) – Medial superior frontal-L (5) – Medial orbitofrontal-R (4) – Anterior cingulate-L (3) – Anterior cingulate-R (3) |
|  | 34 | 32 | Medial orbitofrontal-R (10) – Medial superior frontal-R (9) – Anterior cingulate-L (7) – Medial superior frontal-L (5) – Anterior cingulate-R (1) |
|  | 35 | 54 | Anterior cingulate-L (23) – Medial orbitofrontal-R (11) – Medial superior frontal-R (10) – Medial superior frontal-L (7) – Anterior cingulate-R (3) |
|  | 36 | 53 | Anterior cingulate-L (23) – Medial superior frontal-R (12) – Medial superior frontal-L (6) – Medial orbitofrontal-R (6) – Anterior cingulate-R (6) |
|  | 37 | 45 | Anterior cingulate-L (18) – Medial superior frontal-R (15) – Anterior cingulate-R (6) – Medial superior frontal-L (4) – Medial orbitofrontal-R (2) |
|  | 38 | 34 | Medial superior frontal-R (14) – Anterior cingulate-L (9) – Medial superior frontal-L (5) – Anterior cingulate-R (5) – Medial orbitofrontal-R (1) |
|  | 39 | 29 | Medial superior frontal-R (11) – Medial superior frontal-L (7) – Anterior cingulate-L (6) – Anterior cingulate-R (4) – Medial orbitofrontal-R (1) |
|  | 40 | 23 | Anterior cingulate-L (10) – Medial superior frontal-L (5) – Medial superior frontal-R (5) – Anterior cingulate-R (2) – Medial orbitofrontal-R (1) |
|  | 41 | 35 | Medial superior frontal-L (15) – Anterior cingulate-L (10) – Medial superior frontal-R (6) – 2101 (2) – Anterior cingulate-R (2) |
|  | 42 | 43 | Medial superior frontal-L (24) – Anterior cingulate-L (8) – Medial superior frontal-R (6) – Anterior cingulate-R (3) – 2101 (2) |
|  | 43 | 46 | Medial superior frontal-L (20) – Anterior cingulate-L (10) – Medial superior frontal-R (8) – Anterior cingulate-R (6) – 2101 (2) |
|  | 44 | 58 | Anterior cingulate-L (24) – Medial superior frontal-L (14) – Medial superior frontal-R (10) – Anterior cingulate-R (8) – 2101 (2) |
|  | **45** | **59** | Anterior cingulate-L (30) – Medial superior frontal-R (12) – Medial superior frontal-L (8) – Anterior cingulate-R (8) – Medial orbitofrontal-R (1) |
|  | 46 | 30 | Anterior cingulate-L (16) – Medial superior frontal-R (9) – Medial superior frontal-L (2) – Medial orbitofrontal-R (2) – Anterior cingulate-R (1) |
|  | 47 | 26 | Anterior cingulate-L (18) – Medial superior frontal-R (5) – Medial superior frontal-L (2) – Anterior cingulate-R (1) |
| 3 | 44 | 12 | Medial superior frontal-R (12) |
|  | 45 | 20 | Medial superior frontal-R (19) – Superior frontal-R (1) |
|  | **46** | **25** | Medial superior frontal-R (22) – Superior frontal-R (3) |
|  | 47 | 19 | Medial superior frontal-R (17) – Superior frontal-R (2) |
| 4 | 34 | 3 | Inferior orbitofrontal-L (2) – Middle orbitofrontal-L (1) |
|  | 35 | 7 | Inferior orbitofrontal-L (5) – Middle orbitofrontal-L (2) |
|  | 36 | 16 | Inferior orbitofrontal-L (8) – Middle orbitofrontal-L (8) |
|  | **37** | **19** | Inferior orbitofrontal-L (11) – Middle orbitofrontal-L (8) |
|  | 38 | 16 | Inferior orbitofrontal-L (10) – Middle orbitofrontal-L (6) |
|  | 39 | 10 | Inferior orbitofrontal-L (8) – Middle orbitofrontal-L (2) |
| 5 | 35 | 1 | Middle temporal-L (1) |
|  | 36 | 5 | Middle temporal-L (4) – Middle occipital-L (1) |
|  | **37** | **19** | Middle temporal-L (13) – Middle occipital-L (6) |
|  | 38 | 18 | Middle temporal-L (13) – Middle occipital-L (5) |
|  | 39 | 9 | Middle temporal-L (6) – Middle occipital-L (3) |
|  | 40 | 2 | Middle temporal-L (1) – Middle occipital-L (1) |
| 6 | 20 | 2 | Lingual-R (2) |
|  | 21 | 13 | Lingual-R (13) |
|  | **22** | **14** | Lingual-R (14) |
|  | 23 | 12 | Lingual-R (12) |
|  | 24 | 2 | Lingual-R (2) |
|  | 25 | 1 | Lingual-R (1) |
|  | 26 | 1 | 5022 (1) |
| **Group Thalamus** | | | |
| 1 | 26 | 12 | Cerebellum 7b-L (6) – Cerebellum Crus2-L (5) – Cerebellum 8-L (1) |
|  | 27 | 28 | Cerebellum Crus2-L (15) – Cerebellum 7b-L (11) – Cerebellum 8-L (2) |
|  | **28** | **30** | Cerebellum Crus2-L (15) – Cerebellum 7b-L (12) – Cerebellum 8-L (3) |
|  | 29 | 14 | Cerebellum 7b-L (7) – Cerebellum Crus2-L (4) – Cerebellum 8-L (3) |
|  | 30 | 5 | Cerebellum 7b-L (2) – Cerebellum Crus2-L (2) – Cerebellum 8-L (1) |
|  | 31 | 1 | Cerebellum Crus2-L (1) |
| 2 | 34 | 6 | Middle frontal-R (6) |
|  | 35 | 12 | Middle frontal-R (12) |
|  | **36** | **14** | Middle frontal-R (14) |
|  | 37 | 9 | Middle frontal-R (9) |
|  | 38 | 1 | Middle frontal-R (1) |
| 3 | 35 | 3 | Thalamus-L (3) |
|  | 36 | 7 | Thalamus-L (7) |
|  | **37** | **13** | Thalamus-L (13) |
|  | 38 | 12 | Thalamus-L (12) |
|  | 39 | 6 | Thalamus-L (6) |
|  | 40 | 4 | Thalamus-L (4) |
| 4 | **18** | **4** | SMA-L (4) |
|  | 19 | 3 | SMA-L (3) |
|  | 20 | 1 | SMA-L (1) |

Blues lines corresponds to decreased connectivity with the target, while yellow lines correspond to increased connectivity. In brackets values correspond to the number of voxels in the region.

Note: the labelling was based on the AAL atlas.


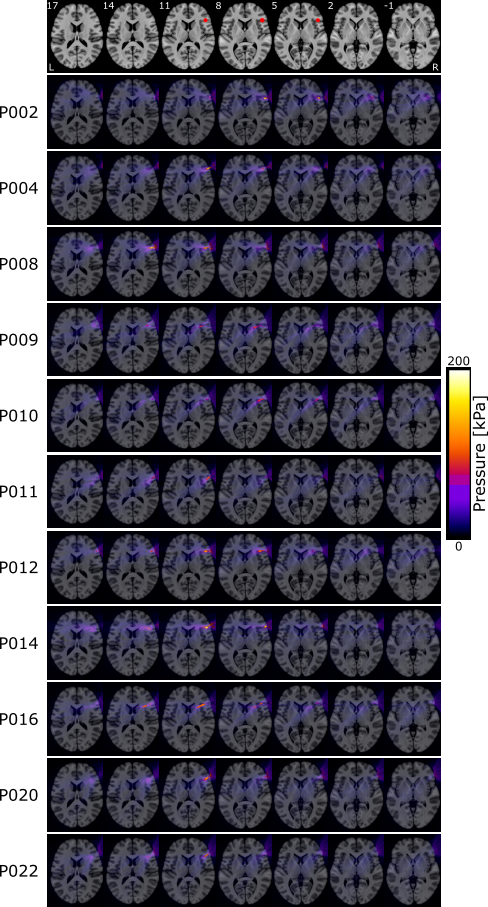


**Figure.A1. Acoustic pressure applied on the target for all participants of the IFC group.**

First row corresponds to the target location. Pressure values were obtained after acoustic simulation performed on k-plan.


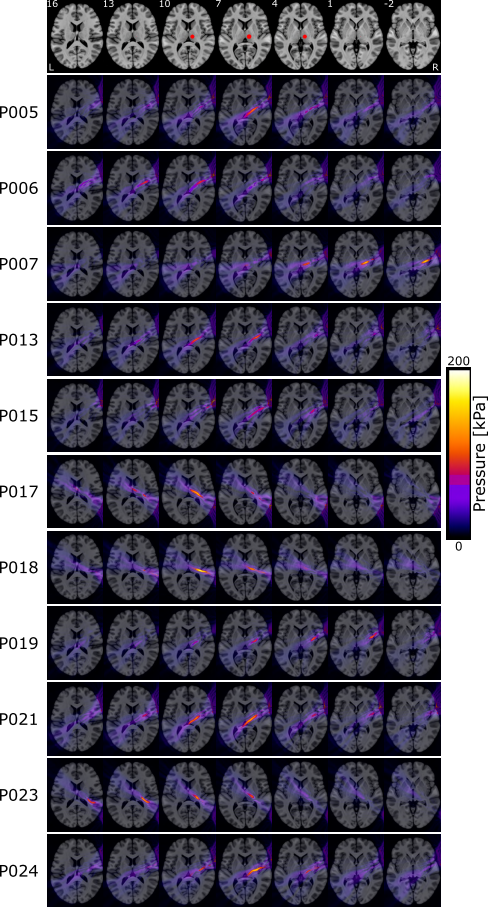


**Figure.A2. Acoustic pressure applied on the target for all participants of the Thalamus group.**

First row corresponds to the target location. Pressure values were obtained after acoustic simulation performed on k-plan.


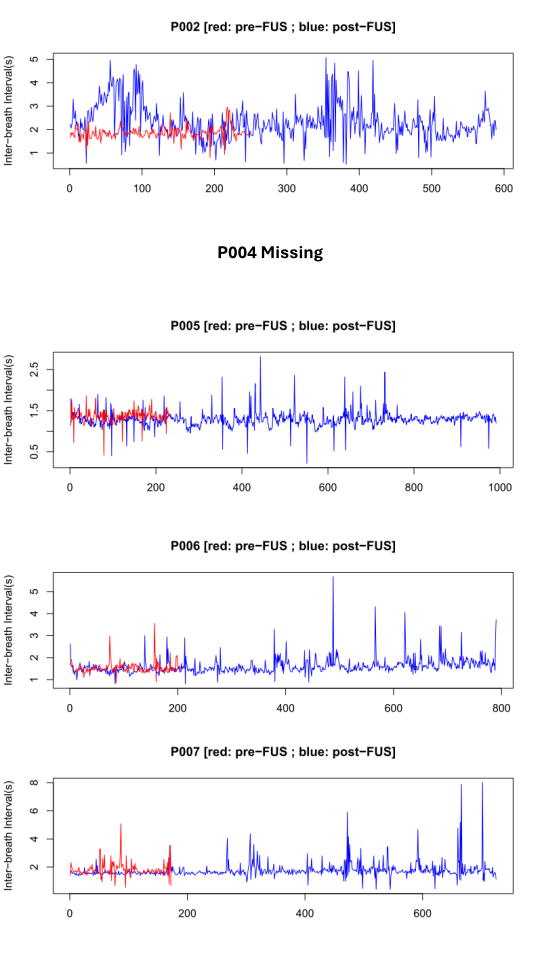


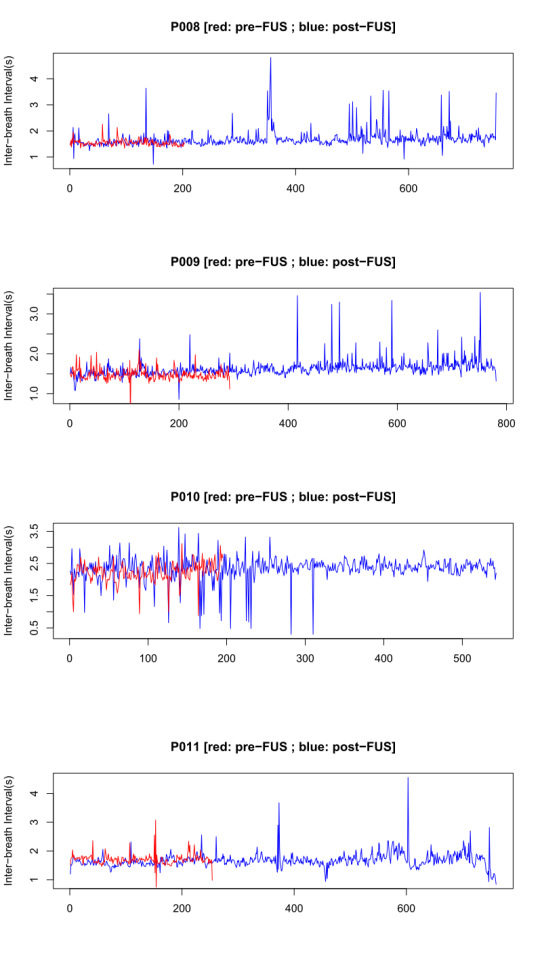


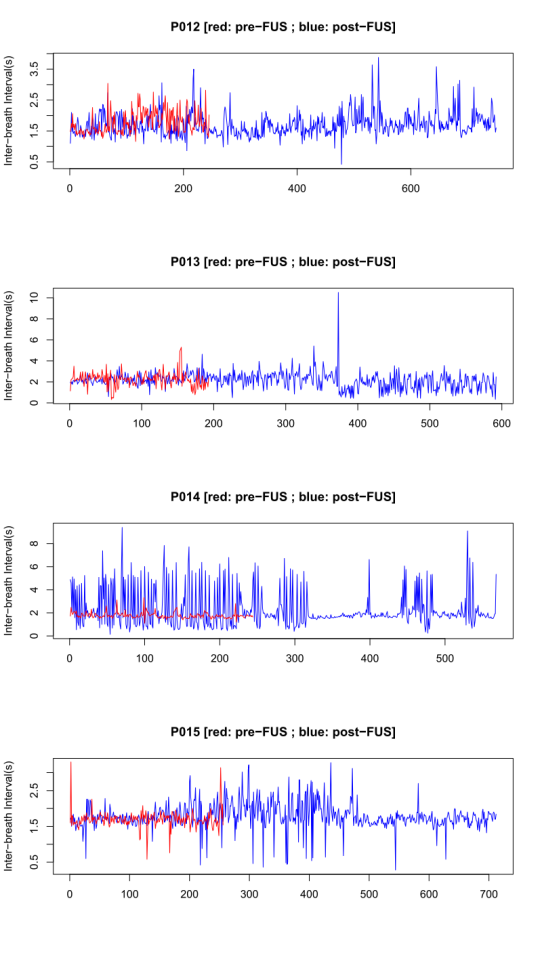


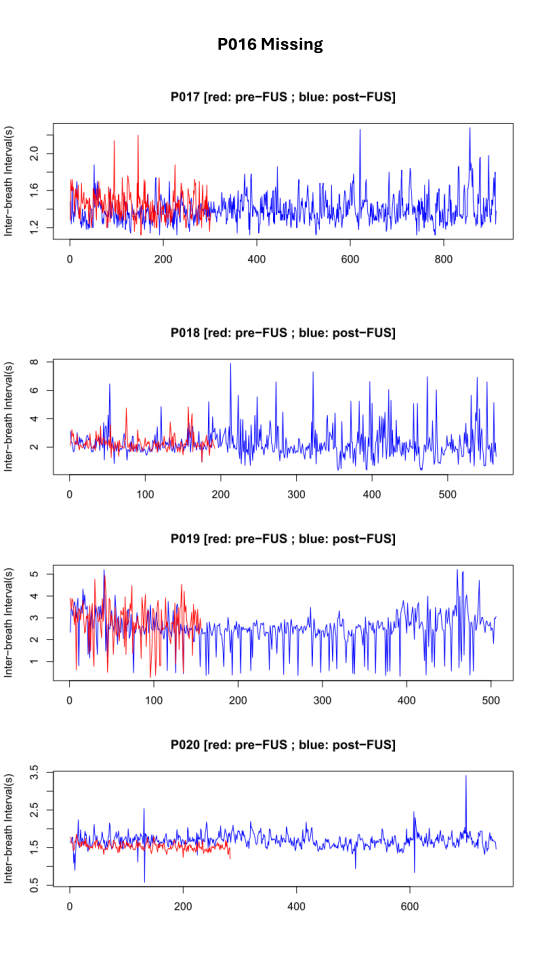


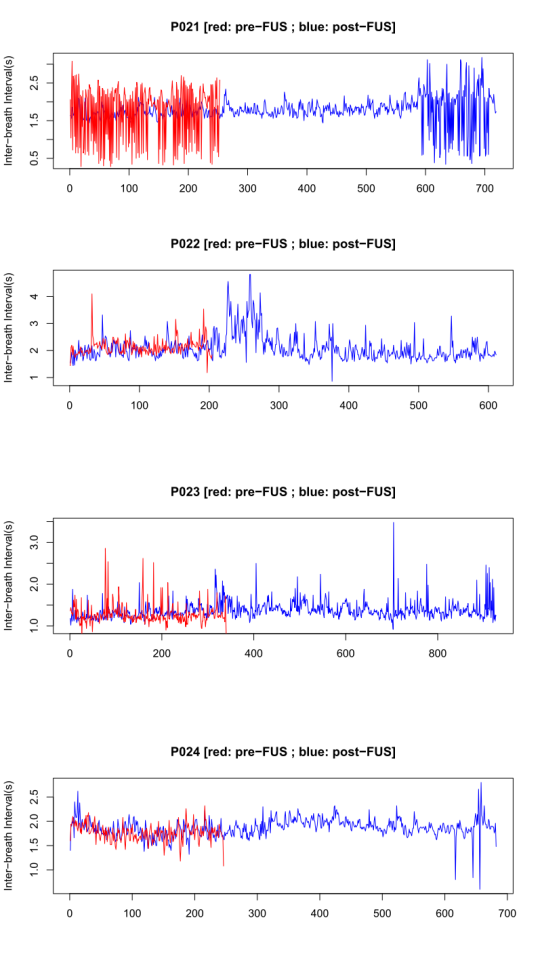


**Figure.A3. Inter-breath intervals obtained during the MRI.**

This analysis shows that none of our participant was sleeping during the MRI acquisitions.


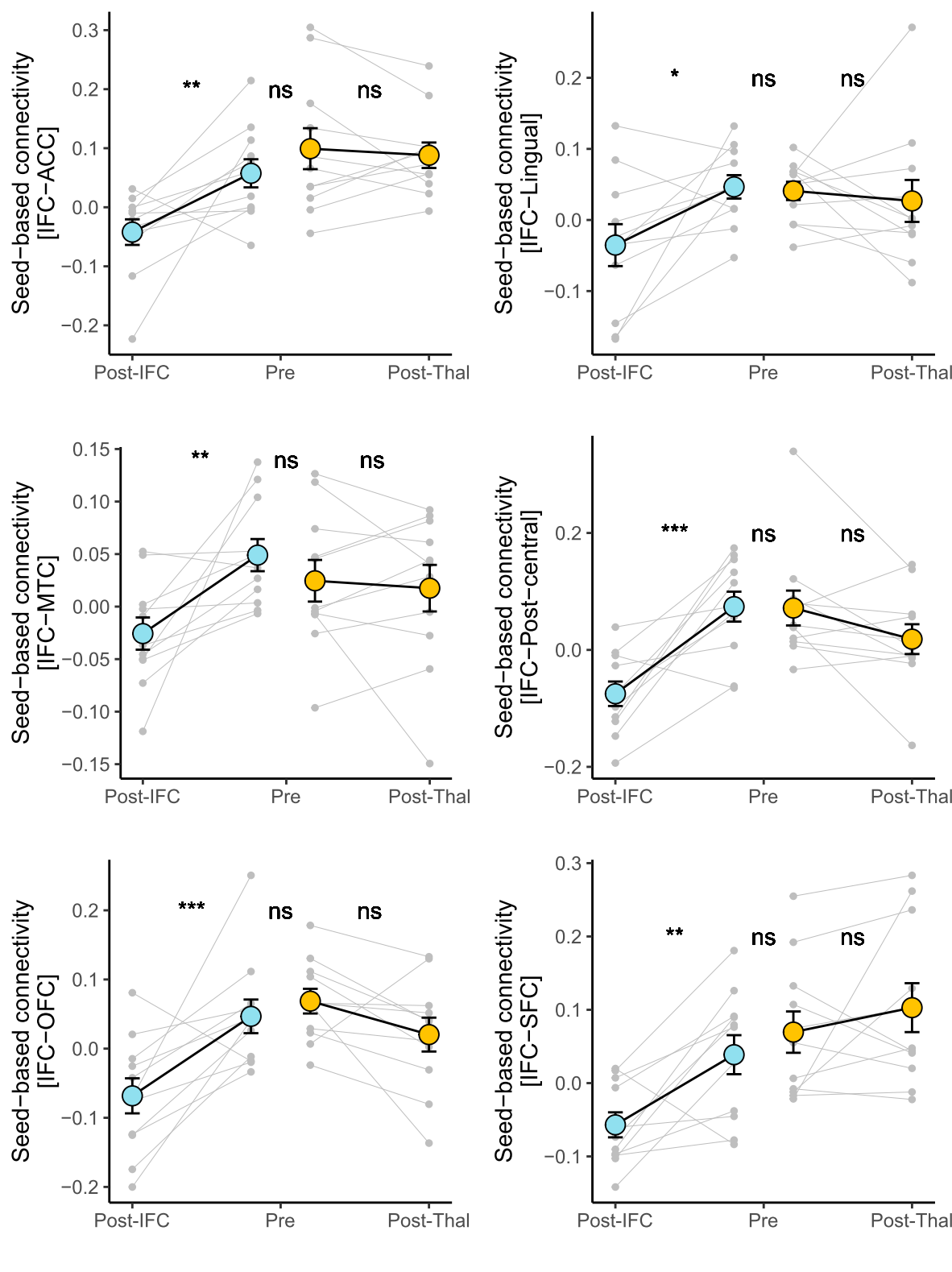


**Figure.A4. Details of the peak of each significant cluster identified following IFC-FUS.**

The post-IFC data are based on the peak in the 4Dimensions (x,y,z,time) while the others were extracted from the static maps. Analyses are based on repeated linear mixed models.


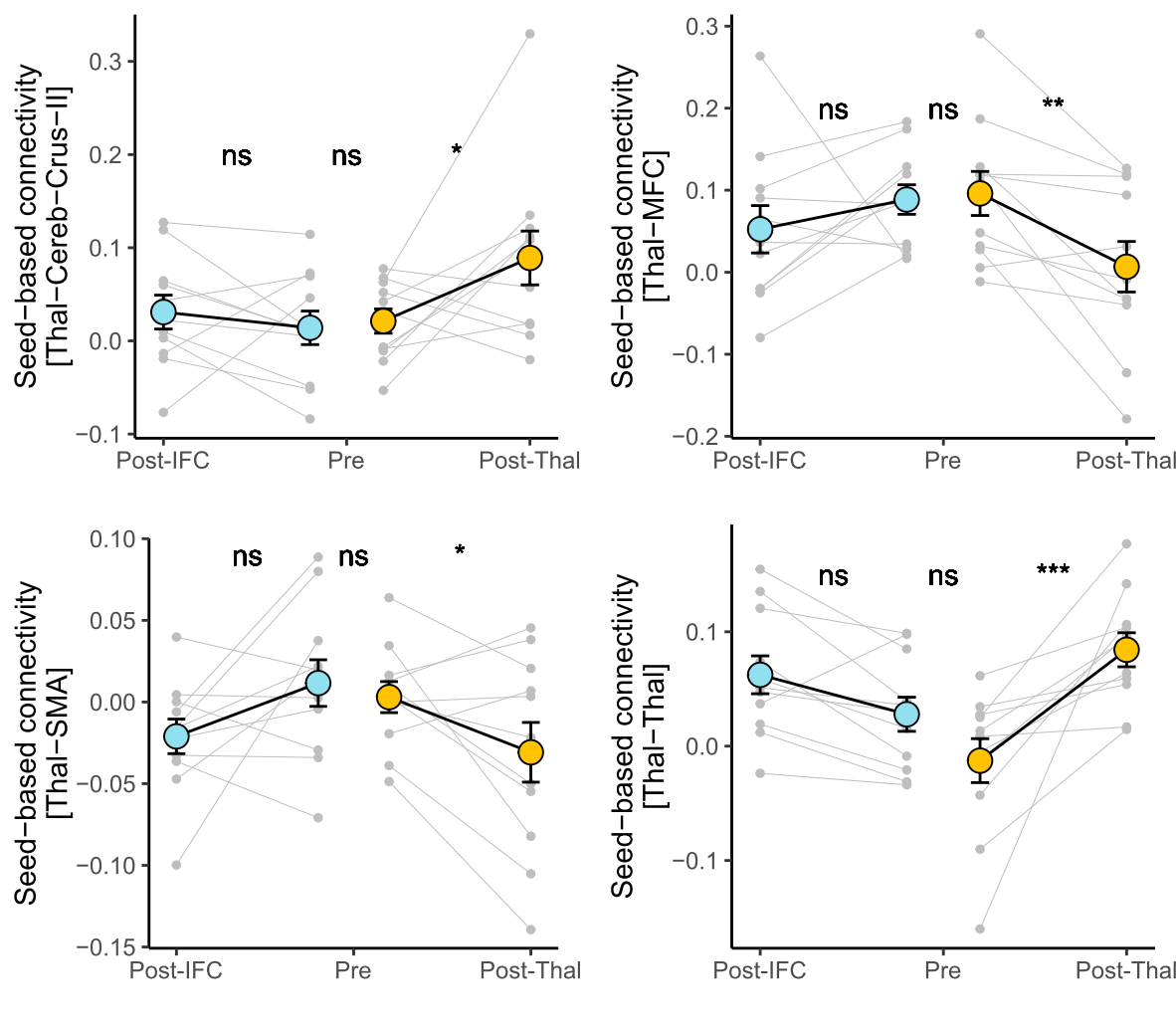


**Figure.A5. Details of the peak of each significant cluster identified following Thal-FUS.**

The post-Thal data are based on the peak in the 4Dimensions (x,y,z,time) while the others were extracted from the static maps. Analyses are based on repeated linear mixed models.
