# Supplementary figures and images for: Dynamic changes in human brain connectivity following ultrasound neuromodulation
Source: Sci Rep. 2024 Dec 3;14:30025. doi: 10.1038/s41598-024-81102-w (PMC11614892; doi:10.1038/s41598-024-81102-w)

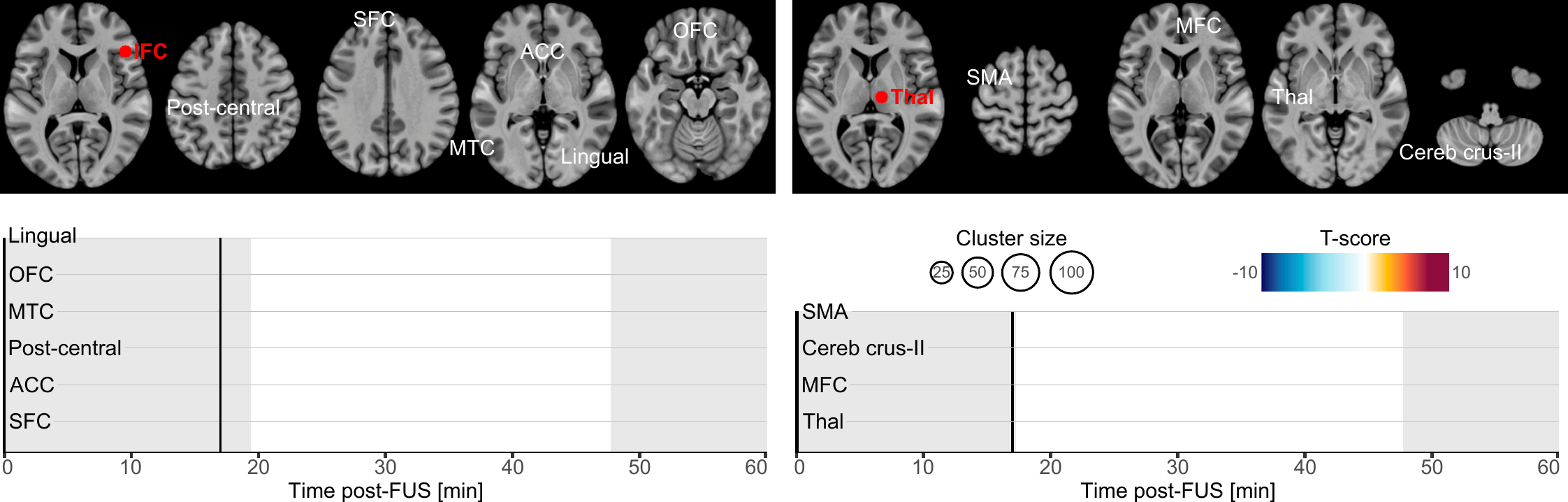

Supplement: Supplementary file 2 — Supplementary Information 2. [file 41598_2024_81102_MOESM2_ESM.gif]
